# Supplementary material for: A novel approach for evaluating contact patterns and risk mitigation strategies for COVID-19 in English primary schools with application of structured expert judgement
Source: R Soc Open Sci. 2021 Jan 27;8(1):201566. doi: 10.1098/rsos.201566 (PMC7890480; doi:10.1098/rsos.201566)
Supplement: Supplement 1. Sparks et al. [file rsos201566supp1.docx]

**SUPPLEMENTARY MATERIALS**

Sparks, R.S.J., Aspinall, W.P., Brooks-Pollock, E., Cooke, R.M., Danon, L., Barclay J., Scarrow J.H. and Cox, J.H. A novel approach for evaluating contact patterns and risk mitigation strategies for COVID-19 in English Primary Schools with application of Structured Expert Judgement**.**

**Section 1. Elicitation Procedures and documents**

Below are the calibration and elicitation questions with covering letters sent to senior school staff. Below are the full calibration and elicitation questions with covering letters sent to senior school staff. The questionnaire has drop-down comments that conditionalize individual questions, which were to be read with additional explanations articulated in the initial guidance/briefing.

*Primary Schools Elicitation Questions*

Our aim is to understand the risks related to a return to school that started in many primary schools on 1^st^ June. Your answers might well be informed by experiencing a typical school week so if your school has not started yet please wait until the end of the first week of restart to complete this elicitation. A major factor in the transmission of an infection is the number of close contacts that a person makes with others and the duration of those contacts. Thus data on contacts can be used to construct a model for potential transmission. In answering the questions below we would like you to cast your mind back to when schools were normal and compare them with now with restricted numbers and school management regimes designed to reduce the chances of an infection spreading.

A contact is defined as a face-to-face conversation within a fixed radius, that lasts for longer than 5 minutes. It is very important that you give us numbers for what actually happens rather than what you hope will happen under ideal conditions and perfect behaviours. Social distancing rules use 2 metres as a guide. Your answers will also help us understand how easy or not it is to follow this rule in a school setting. We infer that it will be quite difficult to manage 2 metres because even for 15 children in a typical sized primary school classroom it will be challenging to achieve this distancing. There may be several strategies you might be adopting; for example using other rooms not normally used for teaching (e.g. school hall), alternating cohorts of pupils on different days of the week and creating small groups of children who work together (bubbles) who are discouraged from interacting with other children during class sessions. The challenges of very young children add to the difficulties. We will ask you some questions that help us understand the strategy you have adopted. For this study we will use a 1 metre distance for our fixed radius. Don’t take this number too literally. What we want is information on how often the famous 2 metre social distancing rule is clearly transgressed for significant amounts of time (5 minutes or more).

For the purposes of our study we will divide up the people in schools into four cohorts: young children, older children, classroom adults and other adults. These are defined as follows:

Cohort 1 Young children of nursery, reception and year 1 age

Cohort 2 Older children in year 2 to year 6 age

Cohort 3. Classroom adults which are teachers and teaching assistants

Cohort 4. Non-classroom adults such as administrative and catering staff.

We appreciate that these are simplifications of reality and will ask you to comment on any significant factors that might affect the risk model by making these simplifications. We are also aware of a wide variety of intake of different age children in different schools, so if you think a question is not relevant to your particular situation (e.g. a junior school with no reception or year1) then just write irrelevant on the question.

Below we ask questions about normal times (before 23 March 2020) and about now (post 23 March 2020) when the numbers of children are reduced and risk mitigation is being practiced in the school. In the questions below we will call these two periods “normal” and “new normal”. We appreciate that every child is different and the number of contacts for a single child might vary considerably. Here we are after an approximate average. However, to get a sense of the range of contacts for individuals we also ask you to judge the highest and lowest number of contacts by a single individual in the cohort. Footnotes help clarify questions further.

[1a] Please describe your strategy to reduce close contacts between pupils (about 50 words). You might want to discriminate between Cohort 1 and 2 above.

[1b] If you use bubbles please describe the number of pupils in a bubble and the approximate spacing between bubbles during class time. The opportunity is given to give separate answers for Cohort 1 and Cohort 2.

Cohort 1. Number per bubble. __________ Spacing between bubbles. __________

Cohort 2. Number per bubble. __________ Spacing between bubbles. __________

[2a] How many people does a typical^[[1]](#footnote-1)^ Cohort 1 child come into face to face contact with (conversation within 1 metre for 5 minutes or more) on a normal school day in a covid free world?

5^th^ percentile _________, 50^th^ percentile _________, 95^th^ percentile __________

[2b] Thinking about the behaviour of individual children^[[2]](#footnote-2)^ give a range of contacts for Cohort 1 children around your central answer to Q2a for normal times.

Most number of daily contacts for a Cohort 1 individual child _____________

Least number of daily contacts for a Cohort 1 individual child _____________

[3a] How many people does a typical Cohort 1 child come into face to face contact with (conversation within 1 metre for 5 minutes or more) on a new normal school day?

5^th^ percentile _________, 50^th^ percentile _________, 95^th^ percentile __________

[3b] Thinking about the behaviour of individual children give a range of contacts for Cohort 1 children around your central answer to Q3a for new normal times.

Most number of daily contacts for a Cohort 1 individual child _____________

Least number of daily contacts for a Cohort 2 individual child _____________

[4] Do you think that there is any significant difference^[[3]](#footnote-3)^ in the contacts made by nursery, reception and year 1 children? No_______ Yes _______

If yes, please indicate the likely difference in contact referenced to reception age children. Put a percentage to indicate a difference (either way); for example you might judge that year 1 children have 80% of the contacts of a reception child.

Nursery _________ Year 1 ___________

[5a] How many people does a typical Cohort 2 child come into face to face contact with (conversation within 1 metre for 5 minutes or more) on a normal school day?

5^th^ percentile _________, 50^th^ percentile _________, 95^th^ percentile __________

[5b] Thinking about the behaviour of individual children, give a range of contacts for Cohort 2 children around your central answer to Q5b for normal times.

Most number of daily contacts for an individual _____________

Least number of daily contacts for an individual _____________

[6a] How many people does a typical Cohort 2 child come into face to face contact with (conversation within 1 metre) on a new normal school day?

5^th^ percentile _________, 50^th^ percentile _________, 95^th^ percentile __________

[6b] Thinking about the behaviour of individual children, give a range of contacts for Cohort 2 children around your central answer to Q6a for new normal times.

Most number of daily contacts for an individual _____________

Least number of daily contacts for an individual _____________

[7] Do you think that there is any difference in the contacts made by Year 2-5 children compared to Year 6^[[4]](#footnote-4)^ No__________. Yes ___________

If yes please indicate the likely difference in contact referenced to year 6 age children. Put a percentage to indicate a difference (either way); for example you might judge that year 2 to 5 children have 120% or 80% of the contacts of a Year 6 child.

Year 2 to 5 ______________

[8a] How many people (both children and other adults) does a Cohort 3 adult come into face to face contact (within 1 metre for 5 minutes or more) with on a normal school day?

5^th^ percentile _________, 50^th^ percentile _________, 95^th^ percentile __________

[8b] Thinking about the behaviour of individual Cohort 3 adults, give a range of contacts for Cohort 3 adults around your central answer to Q8a for normal times.

Most number of daily contacts for an individual. _____________

Least number of daily contacts for an individual _____________

[9a) How many people (both children and other adults) does a Cohort 3 adult come into contact (within 1 metre for 5 minutes or more) with on a new normal school day?

5^th^ percentile _________, 50^th^ percentile _________, 95^th^ percentile __________

[9b] Thinking about the behaviour of individual Cohort 3 adults, give a range of contacts for Cohort 3 adults around your central answer to Q9a for new normal times.

Most number of daily contacts for an individual. _____________

Least number of daily contacts for an individual _____________

[10a] How many people (both adults and children) does a Cohort 4 adult come into face to face contact (within 1 metere for 5 minutes or more) with on a normal school day?

5^th^ percentile _________, 50^th^ percentile _________, 95^th^ percentile __________

[10b] Thinking about the behaviour of individual cohort 4 adults give a range of contacts around your central answer to Q10a for normal times.

Most number of daily contacts for an individual _____________

Least number of daily contacts for an individual _____________

[11a] How many people (both adults and children) does a Cohort 4 adult come into face to face contact (within 1 metre for 5 minutes or more) with on a new normal school day?

5^th^ percentile _________, 50^th^ percentile _________, 95^th^ percentile __________

[11b] Thinking about the behaviour of individual cohort 4 adults give a range of contacts around your central answer to Q16 for new normal times.

Most number of daily contacts for an individual _____________

Least number of daily contacts for an individual _____________

[12] How many children does **an adult (from both Cohort 3 and 4) h**ave physical/face-to-face contact (within 1 metre for 5 minutes or more) during a typical day, in normal times?

5^th^ percentile _________, 50^th^ percentile _________, 95^th^ percentile __________

[13] How many adults does **an adult** **(from both Cohort3 and 4)** have physical/face-to-face contact (within 1 metre for 5 minutes or more) with during a typical day, in normal times?

5^th^ percentile _________, 50^th^ percentile _________, 95^th^ percentile __________

[14] How many children does **an adult** have physical/face-to-face contact (within 1 metre for 5 minutes or more) with during a typical day, in new normal times?

5^th^ percentile _________, 50^th^ percentile _________, 95^th^ percentile __________

[15] How many adults does **an adult** have physical/face-to-face contact (within 1 metre for 5 minutes or more) with during a typical day, in new normal times?

5^th^ percentile _________, 50^th^ percentile _________, 95^th^ percentile __________

[16] **Adherence.** All schools will seek to provide a safe environment following guidelines (e.g. DFE) where feasible and from their own management decisions. Of course some recommendations and procedures may be easier to adhere to than others. We would like your assessment of the extent to which your school is able to follow guidelines. 100% means perfect adherence while 0% means no adherence at all. We have left some space below if you want to make any comments on the any factors that make it difficult to reach 100%

5^th^ percentile _________, 50^th^ percentile _________, 95^th^ percentile __________

**Questions that help us understand better the risk at your school.**

There are other factors that may affect risk in schools that we need to consider so please answer the following questions:

[17] How many children are there whose parents or parents or guardians work in higher risk environments (e.g. care homes and NHS)?

The number of children of essential workers in your school ___________

[18] Describe briefly the cleaning regime in your school using disinfectant (maximum 50 words)

[19] Describe the policy for parents to drop off and pick up their children each day (50 words maximum).

[20] Weather might affect the ability to socially distance and being outdoors is recognised as being less risky than indoors. Estimate the increase in number of contacts as a percentage increase among children as a consequence of bad weather. (e.g. an answer of 30% will indicate the number of contacts increase by this percentage due to play breaks being inside)

5^th^ percentile _________, 50^th^ percentile _________, 95^th^ percentile __________

[21] How many times a day do your children wash their hands? Give a range if applicable.

Number of times ______________ Range_____________

[22] What is your policy if a child or adult staff develops possible COVID19 symptoms (either outside school or during school hours) (50 words maximum)?

[23] What is your policy if a parent or another relative of a child contracts COVID19?

[24} Other Comments. Please make any remarks about additional risk factors that you think should be considered (maximum 100 words).

*Letter sent to experts accompanying elicitation questionnaire*

Dear Colleague,

Thanks so much for persevering with helping this project. We appreciate it particularly as you must all be frantically busy. You will be glad to know that this is the last time that we will be troubling you. However this questionnaire is the most critical for our study and is the real elicitation of you as experts in managing schools under extraordinary circumstances.

I begin with a little background on the science so that you understand why we are eliciting from you these particular questions. The mechanisms of spreading infectious diseases are complex and COVID-19 has some unique features. Mechanisms of transmission include coughing, sneezing, breathing and touching contaminated surfaces. There is mounting evidence for COIVD-19 that transmission is enhanced by indoor settings. The actual transmission events themselves are very hard to observe directly so the proximity of people is used as a proxy. Experiences in many infectious diseases suggest that close contacts and the duration of those contacts are important. Qualitatively face-to-face conversations and touching are thought to be important factors. Thus our questions are aimed at obtaining contact data that can be converted into parameters that go into disease transmission models which are a key part of risk models. Necessarily the contact data has to be simplified.

Surprisingly there are few contact data for children even in settings outside school. There is indeed no data that we are aware of contacts in schools so we are asking you to cast your mind back to pre-COVID19 days when you were running schools as normal. These questions will allow us to assess the relative change in close contacts as you manage your school to minimise contacts to meet social distancing guidelines. The dataset that we acquire with your help will be ground-breaking and may well impact broader issues of disease mitigation. We are also asking you for information to understand better other aspects of risk within primary schools.

Here I would like to introduce you to our colleagues Professor Leon Danon (University of Exeter) and Dr Ellen Brooks Pollock (University of Bristol) who are epidemiologists and experts in using contact data to construct infection transmission models. Leon and Ellen are involved in two of the key science committees that are providing scientific analysis to SAGE. Thus you can be sure that the results of this study will get to the right place quickly.

What approach might you adopt to answering the questions? First we have given a deadline to return the questionnaire of 19 June. This will allow you time to think about the questions and have several schooldays in which to observe your risk mitigation system in action. We are not asking you to spend time trying to make measurements, but on the other hand if you want to share the questions with your staff it could be that they are able to provide some additional observations that help answer some of the questions.

We hope that the elicitation questionnaire will be self-explanatory but to allow us to provide any clarifications and to discuss the issues we are providing a 40 minute webinar at 4 pm on Thursday 11 June. It is not a requirement to attend but simply a chance to discuss the project and further understand the methodology. You will be sent an invite to the webinar in due course. In addition we are happy to answer individual queries by email and to circulate the questions and answers among the group. The email addresses are [Steve.Sparks@bristol.ac.uk](mailto:Steve.Sparks@bristol.ac.uk) and [Willy.Aspinall@bristol.ac.uk](mailto:Willy.Aspinall@bristol.ac.uk).

It will likely take a couple of weeks to process the data once your returns are all in and collated, but we hope that we can send you a report of the preliminary findings in first couple of weeks of July.

Steve Sparks

*Calibration questions for teachers’ elicitation*

**This exercise should take no more than 45 minutes to complete, but it is mentally challenging! *As you will have told countless pupils yourself: Please read the instructions carefully before you start answering*.**

**Purpose:** Based on the responses to the school return head-counts questionnaire, we will build a model for estimating the likely occurrence, and range of uncertainty, for the spread of COVID-19 infections in schools, following partial return on 1 June. The purpose of this exercise is to develop a mathematical scoring basis for combining your collective judgments into a “rational consensus” of uncertainty values for modelling and policy-making purposes.

**Design:** We are using Cooke’s Classical Model, an established technique for combining expert judgments based on statistical measures of expert performance when judging uncertainties [Cooke R.M. 1991 “Experts in Uncertainty”, OUP, 321 pp] <https://en.wikipedia.org/wiki/Structured_expert_judgment:_the_classical_model>

This exercise comprises seven ‘calibration’ questions which, if all are responded to fully, will provide the statistical basis for evaluating your uncertainty judgments from the previous questionnaire. Here, you are asked to give 5th, 50th, and 95th percentile values for your judgment-based probability distributions for the variable identified in each question, and to provide three distinct and different answers to every question to reflect the range of values that are plausible, in your own judgment – not what you think others may think or say.

This last point is key: we are dealing with variables from the first questionnaire for which data do not yet exist, so we need to calibrate your uncertainty judgments from that questionnaire by measuring your uncertainty judgments on these ‘seed’ questions, below. Please note that -- while the facilitator has or will have a ‘true’ value for each seed question -- you are not expected to know exactly the actual value for any question. As far as participants are concerned, it is an uncertain quantity and, given this uncertainty, is best expressed as a credible range.

For any particular seed question, your performance score is not penalised if your uncertainty is large and you express this with a wide credible interval via your three values. However, your scoring is penalised if you attempt to first guess the uncertainty range too narrowly and this range fails to include the true value. Making ‘good’ uncertainty judgments, like this, requires a distinctive and original way of thinking about data and data uncertainty!

*Please note this is not intended to be a test of memory or your ability to google {please don’t be tempted!}, but offers a formalised method for characterising the way participants quantify uncertainties when making judgments in the absence of adequate or reliable data. Note that you are involved as an expert because of your specialist knowledge and experience in school management and as a teacher. However, this is not a test of that knowledge per se.*

***Lastly, this exercise is conducted confidentially and, as far as others are concerned, anonymously: your judgments and calibration score will not be attributable to you personally.***

**Your actions:**

*Please give your lower, median and upper credible interval values which, in your judgment, should capture the actual number as reliably as possible (i.e. approx. 5^th^, 50^th^ and 95^th^ percentile values); your 50^th^ percentile value (i.e. median) need not be central within your 5^th^ – 95^th^ percentile range. Bear in mind there is a 1-in-20 chance that the actual value may fall below your lower marker value, and a 1-in-20 chance that it may be above the upper marker -- so, while it is not out-of-the-question, you should be quite surprised to learn that you “missed the dart board” on certain questions.*

SQ1 DfE data on school pupils in England 2018-19: the national average percentage of overall absence (authorised and unauthorised) for the full 2018/19 academic year was 4.6%.

What was the highest percentage overall absence in any single school?

………………………………… ………………………………… ..……………………………..

lower credible interval most plausible true value upper credible interval

(5^th^ percentile) (50^th^ percentile = median) (95^th^ percentile)

SQ2 DfE collated reasons for pupil absence in schools in England for 2009/2010. One category is “Authorised illness (not medical or dental appointments)”. This reason constituted what percentage of absences in Year 6 pupils?

………………………………… ………………………………… ..……………………………..

lower credible interval most plausible true value upper credible interval

(5^th^ percentile) (50^th^ percentile = median) (95^th^ percentile)

SQ3 A survey of self-reported contact networks in Year 7 groups in four UK secondary schools over 4 rounds of data collection. One school had low returns due to lack of parental consent.

In the other three schools, two co-eds in London and West Sussex and a single-sex school in London, what overall percentage of the Year 7 pupils participated in at least one round of data collection?

………………………………… ………………………………… ..……………………………..

lower credible interval most plausible true value upper credible interval

(5^th^ percentile) (50^th^ percentile = median) (95^th^ percentile)

SQ4 The same survey collected contact data over four rounds between January and June 2015, in the four schools. Students were asked to list up to six other students in Year 7 at their school with whom they spend the most time. (Limiting to no more than six named contacts follows from a previous study: with no upper limit there is a risk of deliberate over-reporting due to ‘competitive naming’ via peer pressure). A total of 460 individual Year 7 participants completed 1,254 surveys nominating their most frequent personal contacts. The survey question had a limit: no more than six others could be listed as contacts.

With that constraint, what was the overall total number of identifiable different individuals reported as contacts by the survey respondents in the four schools?

………………………………… ………………………………… ..……………………………..

lower credible interval most plausible true value upper credible interval

(5^th^ percentile) (50^th^ percentile = median) (95^th^ percentile)

SQ5 A different social contact survey was conducted for GB to improve understanding of the nature of individual-level social contact patterns that are relevant to the potential spread of infections. Included were school-age children (age 4 – 17 years). The data were converted into “person-hours of contact” per day, i.e. each child reported their own number of contact persons in the day, and this is multiplied by the total time spent in such contacts. For example, a child who accumulated 4 hours of contact time with 5 contacts would be recorded as having 20 person-hours of contact on the day in question.

The survey reported that these school children (age 4 – 17 years) had an average 39 person-hours of contact on a non-school day. What was the average number of person-hours contact on school days?

………………………………… ………………………………… ..……………………………..

lower credible interval most plausible true value upper credible interval

(5^th^ percentile) (50^th^ percentile = median) (95^th^ percentile)

SQ6 Between 30 March and 4 April 2020, a retrospective cohort study was conducted among pupils, their parents and siblings, as well as teachers and non-teaching staff, for a high-school located in northern France. 38.3% of (240) pupils tested positive for SARS-CoV-2 serological antibodies.

There were 27 non-teaching school staff in the cohort. What percentage of these tested positive?

………………………………… ………………………………… ..……………………………..

lower credible interval most plausible true value upper credible interval

(5^th^ percentile) (50^th^ percentile = median) (95^th^ percentile)

*Finally, a tough question, requiring serious thought! If clarification is needed, please contact <willy.aspinall@bristol.ac.uk>*

SQ7 Three alternative closure strategies for dealing with a flu-like disease outbreak within a primary school’s premises might be: class closure; Year group closure or school-wide closure. In order to estimate the effectiveness of each of these strategies for limiting infections, a numerical model based on contact data was used to simulate the spread of infection among pupils. The model assumed that if a pre-agreed number count of symptomatic infectious individuals in any one class in the school was encountered, this would be the trigger for activating a response strategy. As a benchmark, the model predicts that if two or more symptomatic children were detected in a class and simply isolated, and that none of the three closure steps is taken, then there is a 34.6% probability (about 1-in-3 chance) that the school could expect more than 10% of its pupils to become infected.

If two symptomatic students were detected in one class of a primary school and the whole school is closed for three days, what percentage probability does this **model predict** for the school experiencing more than 10% of pupils being infected?

………………………………… ………………………………… ..……………………………..

lower credible interval most plausible true value upper credible interval

(5^th^ percentile) (50^th^ percentile = median) (95^th^ percentile)

**Section 2. Structured interviews**

This section describes the scoping, organization of the structured interviews with six teachers from our study group. Interviews carried out by Professor J. Barclay

Introduction – explain who we are – purpose of interview is to understand a little bit more about the thought processes and mental models that informed the numerical answers given. This will help the research team to interpret the results that they have obtained from the study in terms of views about contact between children and adults in the School setting.

Question Guide…(where questions are answered in conversation please skip)

Question 1. *Most of the questions were in two parts and dealt with pre-COVID and COVID behaviour.*

*The first part usually asked about the behaviour of a typical individual in any year group, and the second was more often thinking about the range across a group.? Can you tell me about how you came about the answer for a typical child.*

b. *So what were you thinking about with the children when you were thinking about how that varied?*

Question 2. *Related to this (if unanswered by question 1 discussion) when thinking about your 95^th^ or 50^th^ centile levels of contact did you tend to think these were governed by children having larger group work in class or in playground or lots of relatively short-lived single contacts? Were you factoring in behaviour at breaks etc?*

Question 3: so do you broadly think your uncertainty reflected uncertainty in the number of contacts for each question or a wide range in likely behaviours between children and teachers, or on a day to day basis?

Question 4: Can you talk a little bit about your rationale in moving from the answers you gave for normal times to thinking about new normal.

Question 5: when thinking about ‘contacts’ between the children in your school what differences do you perceive between Year 6 and Year 1 children in terms of how they typically conduct work in the classroom and how they typically play during break time and during PE?

5b. Is this a gradual change across the years, or is there a moment when things alter more perceptibly?

6. Did you apply similar rationales in thinking through the questions about teacher contacts?

6b What did you imagine as the job description/daily routine of those who perhaps represented the 5^th^ and 95^th^ centile of your answers

6c What did you take into account when you provided the new normal values for this group?

7. Based on experience how did you visualise poor weather affecting contacts?

8. Have you subsequently had to deal with children with COVID or thought through some more how to cope with that?

[prompts: have you a protocol for contacting other children in their bubble, or for dealing with children or teachers who contract COVID outside of school?}

9. Only if Y to 9 – what happened? Did the reality match the protocol?

**Interview 1.**

Question 1. *Most of the questions were in two parts and dealt with preCOVID and COVID behaviour.*

*The first part usually asked about the behaviour of a typical individual in any year group, and the second was more often thinking about the range across a group.? Can you tell me about how you came about the answer for a typical child?*

(prompts, what is a typical child – is it constructed across year group or school, or by imagining *yourself in a classroom).*

**I thought about the average child who would more or less follow instructions typical for their age group. I’ve taught across the age ranges R, Y1 and Y6. Thought about the layout of the classrooms timetable staffing and the equipment. Discussed the typical days and habits with the teachers. This school has a lot of children in Key worker bubbles (45) and so with R and Y1 which are 42 and 40 they had the school used up with 15 kid bubbles in their nine classrooms. So Y6 only came back this last week.**

**Thought across the whole teaching day lunches and playtimes and the different types of activities they might do in the classroom. The message in this school is within the bubble try not to stress too much about contact within bubbles, but not contact between bubbles.**

*So what were you thinking about with the children when you were thinking about how that varied?*

**In COVID times there is really much less variation for the children within their bubble. By tailoring** **the day to the COVID approach the day-to-day variance in contact is going down. The only time things vary from day to day is with wet weather. And really the weather has not been very wet so this did not have a big impact on thinking (see answer to Q8 too).**

**Also thought through how this varied from child to child for the cohort answer. This variance is very dependent on the age of the child, but this is mainly what she thought about (although did also think about how uncertain her answering was when she thought about it). So for example younger kids are more tactile and some of them really want a cuddle or they might be more likely to have accidents that encourage contact so though about that.**

**What she thought a lot about was what constituted face-to-face with > 5 m at < 1 m this was a recurring theme throughout her thinking. This is a lot less frequent than the rapid interactions typical of working with small kids who might come up for a quick hug and then run off again. So she logically thought through the activities and how it would work in the classroom (example used was her thought process around reading) every child read to a teacher for more than 5 minutes but side-to-side but she thought that through and thought really it’s not possible for the kid and teacher not to look at each other and probably that amounted to 5 minutes over reading so that counted as one contact). So very careful thought process.**

**She then made the point that the older kids in the playground are actually more likely to have more contact because they might stand in groups and chat to each other whereas the wee ones just bomb around and interact but really in parallel and not close so nothing that might constitute a contact.**

**This varies though – for example ‘the footballer’s would play all the time but have no ‘contact’. something in the past they always did was bring stuff from home but now they do not do that – and actually that has encouraged much more active play and less chat – so with e.g. hoops – and all of this actually keeps them naturally a bit more distant.**

**All of this was considered in the range of kid contacts for each year!**

(prompt: so for example did you think there was an even distribution between the different ways that children behaved – so for example some prefer to play in largish groups together, some form strong pairs, some tend to seek out adult company for reassurance at differing times?)

Question 2. *Related to this (if unanswered by question 1 discussion) when thinking about your 95^th^ or 50^th^ centile levels of contact did you tend to think these were governed by children having larger group work in class or in playground or lots of relatively short-lived single contacts? Were you factoring in behaviour at breaks etc?*

*If yes, can you just briefly run through how children are managed during breaks.*

**See above**

Question 3: so do you broadly think your uncertainty reflected uncertainty in the number of contacts for each question or a wide range in likely behaviours between children and teachers, or on a day to day basis?

**Wide range of behaviour with a little thinking of uncertainty see above – and answer coming about difficulties in COVID times.**

Question 3: Can you talk a little bit about your rationale in moving from the answers you gave for normal times to thinking about new normal.

**Ran through and imagined the working day under both conditions. How many different activities would a child have in each day that counted as a contact. Anytime they would, for example, be in a small group doing work facing each other that counted as contact. Playtime contact – and have to say its so much harder to estimate in normal times because behaviour and contact varies so much more. In the new normal they are very contained with very controlled contacts. No contact with the midday supervisors but in old times there would be a huge variation between how kids would have contact at lunch so for example R and Y1 would all have dinners because its free – that is more contact with each other and the supervisors but kids having packed lunches are spread apart more – this happens more in years 3-6. It was so much harder to feel confident about the numbers thus from old times because these are all controlled now but varied then. Also there would be large variation with a child’s character. You imagine a social child, one of them would pretty much have contact with every child in their class and likely they are more confident so would go and hang out and have confidence with siblings and friends at breaktimes. They’d probably go and see people in the office. Much easier to estimate now because they just would not do that!**

**Really the hardest thing of all in thinking through normal times is how many of these many interactions would count (as > 5 min, < 1 m).**

[what do you think the predominant control was on the reduction in contacts).

Question 4: when thinking about ‘contacts’ between the children in your school what differences do you perceive between Year 6 and Year 1 children in terms of how they typically conduct work in the classroom and how they typically play during break time and during PE?

**Mainly already answered. But also said R and Y1 very similar but by Y3 there is no afternoon play and Y4 seated as would be in COVID times (in rows apart). Really though she thought older children might have more counting contact during breaks, but again this is very dependent on the child; the sporty kids have less contact because they are running about more. The quieter kids would perhaps have more contact but with less individuals.**

4b. Is this a gradual change across the years, or is there a moment when things alter more perceptibly?

A range in behaviours in every year and a gradual change.

5. Did you apply similar rationales in thinking through the questions about teacher contacts?

**Yes. Again it was so much easier for working it out for now and much harder to quantify with normal times. At the moment with T and TAs’ every group has 2 adults and the bubble sticks to itself and the kids have no contact with other staff. So just thought through what that adult would do in a day and asked them (how many times they would sit at a table close to a small group of kids (- intimated about 4 or 5 times a day during teaching). If the teacher is standing in front of a whole class this is less likely to count. The TAs are more likely to have contact but a stronger focus on less kids (guided reading etc).**

**In normal times it’s a nightmare. How do you count assembly (we are ‘packed in like sardines’ so she recounted it). Then there is the daily interventions – most of us have perhaps two kids we spend concentrated time with – so that also count. She thought it through typically for each teaching year. And how that varied as the year group and the teacher varied.**

**In thinking through the typical and cohort – she imagined an average kind of teacher as typical and the more specialised TA as less typical (5 and 95 centile).**

**Then revealed how our contact counting also accumulated. Any interaction if continuous was 1 but if it was a contact then a break and then a new contact – she counted that as two as they could have got infected or become infectious in the interim.**

[to clarify – was the job profile of the person who informed your 50^th^ centile answer, and that of your 5^th^ centile different for example}

Supplementary questions. Who were you thinking of as non-teaching staff? Is there an average role or job profile in this group?

**Non-teaching staff = office, cleaners and the cook. And two midday supervisors at moment. She thought these through by starting with the extremes. The cook should have no contacts (as only adults and they should physically distance) and then some of the others more. But, she was a bit ‘secret’ squirrel’ and observed – no-one is totally sticking to that (adult-adult contact). It’s hard and a bit boring to be good. And then how do you remember what a metre is? So she factored in this non-compliance into her variance too – what they less contact person should be doing and the reality.**

**This would be massively different in normal times. She said really it made her realise the distancing has had a huge impact on this group. The office would have had the most contact with parents/children – they usually would have the most face-to-face of everyone in the office. It’s a big difference and that is a hard difference.**

**Then a lot of adult –adult interaction went in the staff room – and spending lunchtimes with the children further reduces that.**

Prompts: what did you imagine as the job description/daily routine of those who perhaps represented the 5^th^ and 95^th^ centile of your answers

What did you take into account when you provided the new normal values for this group?

**See above – range of contacts (but did consider uncertainty particularly with old times!)**

8. Based on experience how did you visualise poor weather affecting contacts?

**Yes. This would have a big impact but in making her answers she only counted two weather affected days so did not factor it in much – it would in the future as they really relied on outside play to keep the weather effects down and also always had windows open. Bad weather routine here would push daily contacts right up – kids much more likely to interact and talk to kids they might not usually during breaks.**

9. Have you subsequently had to deal with children with COVID or through through some more how to cope with that?

**2 had test from keyworker parents contact. Both –ve. Drill is to isolate until test result then if +ve the bubble would get shut down**.

**With the return to school she anticipates the bubbles will become class sized (30) and that in the playground will become whole year group (2 bubbles). She anticipated that this would then mean whole class home but would hope not both classes if contact outside… but would expect and act on PHE advice.**

[prompts: have you a protocol for contacting other children in their bubble, or for dealing with children or teachers who contract COVID outside of school?}

10. Only if Y to 9 – what happened? Did the reality match the protocol?

**Interview 2.**

Preamble

**Found the questions to be really hard, as thinking through interactions and the extent of the interaction were hard. While doing the questionnaire she asked direct questions of different members of staff (said to cleaning staff – ‘do you see the kids, do you have a chat with them?’), and thought through the risk proclivities of her various members of staff.**

**Also told me that because of a meeting in school she missed the online meeting and explanation and so felt to some extent she was ‘making it up’ how she thought it ought to be.**

Question 1. *Most of the questions were in two parts and dealt with pre-COVID and COVID behaviour.*

*The first part usually asked about the behaviour of a typical individual in any year group, and the second was more often thinking about the range across a group.? Can you tell me about how you came about the answer for a typical child.*

**On going back into school she was actually in a couple of bubbles that changed through time. The bubbles were very important, and she had experience of bubbles across several year groups so this really informed her thinking, actually thinking through her day in the bubble and how that varied from day to day. The bubbles in this school are really important and form a tight knit community. The kids really stick with their bubble as do the teachers. About 80% of her day is being in the bubble.**

**She really incorporated the different stages of the school day into her thinking playtimes, lunchtime (and even mentioned thinking about a typical procedure when a child injures themselves). At the beginning there was a little bit more cross-contamination of the bubbles but the separation of that bubble (up to 15 children) is important now and was important to her answers. Creating the larger bubbles with more than one teacher facilitated this intra bubble strictness (she said this bit later).**

**Very strong recognition of the mental health importance of coming back to school and its value to children (mentioned and discussed several times). Strong emphasis on more school.**

(prompts, what is a typical child – is it constructed across year group or school, or by imagining *yourself in a classroom).*

*So what were you thinking about with the children when you were thinking about how that varied?*

**Really her particular school has very few behavioural issues within it – and so because of that and the tight knit natures of the bubble children were all behaving more typically – so less variation at the moment. However, this variance was much more informed by how school days and the encounters of children might vary from day to day than within cohort variance. So the range of things experienced by them as a group.**

**To some extent there is a lot less variance for the teacher and the children – to keep the bubble isolated and broadly experiencing the same things together.**

**Later she did also remark that it was also reflectance of uncertainty in how to answer. These questions were very difficult to put a ‘number on it’ and was mentioned a couple of times in this context thinking through the ‘5 minute contact’.**

(prompt: so for example did you think there was an even distribution between the different ways that children behaved – so for example some prefer to play in largish groups together, some form strong pairs, some tend to seek out adult company for reassurance at differing times?)

Question 2. *Related to this (if unanswered by question 1 discussion) when thinking about your 95^th^ or 50^th^ centile levels of contact did you tend to think these were governed by children having larger group work in class or in playground or lots of relatively short-lived single contacts? Were you factoring in behaviour at breaks etc?*

*If yes, can you just briefly run through how children are managed during breaks.*

**See answer to Question 1 – mainly pinned on the range of encounters day to day and some uncertainty in how to answer the question.**

Question 3: so do you broadly think your uncertainty reflected uncertainty in the number of contacts for each question or a wide range in likely behaviours between children and teachers, or on a day to day basis?

**Day to day variance**

Question 3: Can you talk a little bit about your rationale in moving from the answers you gave for normal times to thinking about new normal.

**Mentally walked thought the typical day for her children and answered then - imagined an old day – which she said was harder because right now they are ‘living with the bubbles’ but she did think back through that day, but then also thought through in particular places where lots of contact would be happening to add them on. The example she gave was pre-lockdown the kids would really talk to the dinner ladies. Now they come in and have the same seats they sit in all day for lunch and it is brought to them by their bubble teachers.**

[what do you think the predominant control was on the reduction in contacts).

Question 4: when thinking about ‘contacts’ between the children in your school what differences do you perceive between Year 6 and Year 1 children in terms of how they typically conduct work in the classroom and how they typically play during break time and during PE?

4b. Is this a gradual change across the years, or is there a moment when things alter more perceptibly?

**Yes, KS1 and KS2 are very different. At KS1 age the kids are still at the stage where they are lying down on the floor to rest and spend a lot more classroom time playing and really don’t bother socially distancing in the playground. KS2 are better at social distancing and it’s a more formal learning environment, however at playtimes they are not really socially distancing within their bubbles either.**

**How this is working in classroom was also dependent on individual teacher attitudes too, however. Although her attitude was ‘if I get it I get it’ but e.g Year 6 teacher more afraid and this would have an independent influence on contact in the classroom. She thought about that too in her answers – the role of individual teacher attitudes and the variance that might cause. She did also ask questions of her staff about this.**

**This transition to more formal socially distant learning is gradual there is not an abrupt transition from KS1 to KS2.**

5. Did you apply similar rationales in thinking through the questions about teacher contacts?

[to clarify – was the job profile of the person who informed your 50^th^ Centile answer, and that of your 5^th^ centile different for example}

**I know my teachers quite well and based it on the degree of caution in teaching. Other staff are cleaners, receptionists and dinner ladies (who are effectively all the same people at the moment!). Typical staff member was a very hard one thus to think of because of the range of behaviours and attitudes – but it was largely governed by that range in behaviour (the centiles) rather than uncertainty in the answer. (see below).**

**At this point she made the point that the questions echoed how one teaches – ‘you plan for the middle’ and then think about how you deal with and understand the extremes (in her example the very bright kids and the ones with special needs).**

Supplementary questions. Who were you thinking of as non-teaching staff? Is there an average role or job profile in this group?

Prompts: what did you imagine as the job description/daily routine of those who perhaps represented the 5^th^ and 95^th^ centile of your answers

What did you take into account when you provided the new normal values for this group?

**This involved much more thinking about where the children were not going because they were restricted to their bubbles at all times. No little errands, no time talking to people in the office – but there would be lots of that for them in normal times.**

8. Based on experience how did you visualise poor weather affecting contacts?

For the bubbles it just influenced where the contact was happening not how much. Also mentioned they keep classrooms well ventilated windows open etc.

9. Have you subsequently had to deal with children with COVID or through some more how to cope with that?

**One child and sibling kept at home until had test result (negative). Her daughter (Y10) was also tested so she self-isolated, but while waiting for a result on the test was isolated. Bubble goes as soon as positive. This has not happened but is well thought through – and reason for the really strong focus on the bubble. Things are changing all the time however so its hard to keep up with planning! Concerns with geographic location and introduction of COVID over summer via tourists.**

[prompts: have you a protocol for contacting other children in their bubble, or for dealing with children or teachers who contract COVID outside of school?}

10. Only if Y to 9 – what happened? Did the reality match the protocol?

**Interview 3.**

Question 1. *Most of the questions were in two parts and dealt with preCOVID and COVID behaviour.*

**The first part usually asked about the behaviour of a typical individual in any year group, and the second was more often thinking about the range across a group? Can you tell me about how you came about the answer for a typical child.**

**For a typical child he considered someone normal for that setting. This is a normal child in their schools context where they have quite a range of behaviours. The normal child here would perhaps not be the same as a normal child for example going to school in Clifton in Bristol.**

**He then made a mental model of the school day for that child (relevant to age group) considering school, breaks and playtimes and mealtimes. He did not include parents as contacts.**

**For looking at the 5^th^ and 95^th^ centile he tended to think about less typical days where something varied in that routine.**

**Essentially the same procedure when thinking about the whole group but this time the average behaviour factored in some of the less normal behaviour so it was a total of all the children ‘averaged’. Some of these children are very challenging some of them need restraints.**

**He remarked that the normal and new normal were very different to normal times the atypical days – so for example in normal times a less usual day would be a school trip or when they had a group of visitors to the school – this does not happen – they are strict with their bubbles and so there is less variance.**

**He did consider some uncertainty into the answers but his approach was to think through a typical pattern and his uncertainty around that and then consistently apply it in a similar way to all the questions.**

**The school was very quick off the mark and had R, 1 and Year 6 all back – so he could see the children to understand and think through his answers – he could see what was happening in practice.**

**Even during normal times he is a peripatetic head so would have a good idea – and he did also do some year six teaching so had a lot of evidence to think through.**

(prompts, what is a typical child – is it constructed across year group or school, or by imagining *yourself in a classroom).*

*So what were you thinking about with the children when you were thinking about how that varied?*

(prompt: so for example did you think there was an even distribution between the different ways that children behaved – so for example some prefer to play in largish groups together, some form strong pairs, some tend to seek out adult company for reassurance at differing times?)

Question 2. *Related to this (if unanswered by question 1 discussion) when thinking about your 95^th^ or 50^th^ centile levels of contact did you tend to think these were governed by children having larger group work in class or in playground or lots of relatively short-lived single contacts? Were you factoring in behaviour at breaks etc?*

*If yes, can you just briefly run through how children are managed during breaks.*

**See above**

Question 3: so do you broadly think your uncertainty reflected uncertainty in the number of contacts for each question or a wide range in likely behaviours between children and teachers, or on a day to day basis?

Question 3: Can you talk a little bit about your rationale in moving from the answers you gave for normal times to thinking about new normal.

[what do you think the predominant control was on the reduction in contacts).

**See above**

Question 4: when thinking about ‘contacts’ between the children in your school what differences do you perceive between Year 6 and Year 1 children in terms of how they typically conduct work in the classroom and how they typically play during break time and during PE?

4b. Is this a gradual change across the years, or is there a moment when things alter more perceptibly?

**For year 6 to Year 1 the key difference is the lack of emotional intelligence that the younger group have. You could come in the door of a Year 1 and then they would run up and hug you. This happened hundreds of times. Some of those children are not aware and just extremely tactile. Year 6 were very aware and mature and would pick up on the rules they had to adhere to and do that. There would be some differences the children are from very different background with very different life histories so that gives rise to some of the variability within any one year group.**

**In the playground? (prompt from me). Year 6 are really good at keeping their distance – and also would respond well to use creating games and activities for them that kept them apart the yr1s were less able to cope with that and so struggle to keep apart. The government realised largely this would be the case, and the staff did too and were comfortable with that.**

**In terms of gradual that is something harder to answer. What I could see in front of me were nursery, Year 1 and Year 6, so its hard to visualise how that might change with only the evidence of the Keyworkers children but I would guess it would be gradual.**

5. Did you apply similar rationales in thinking through the questions about teacher contacts?

[to clarify – was the job profile of the person who informed your 50^th^ Centile answer, and that of your 5^th^ centile different for example}

**Broadly, yes. The teachers are much more typical though (less differences) they all had pretty similar comfort levels in this school. The main difference is the age of the children they would be in front of (Year 6 very little direct contact in this way with kids, Year 1 and younger lots of encounters). At the school some of the lessons are conceived by the teacher and delivered by the TA so the teacher has contact with the special learners, so they are also quite similar too in patterns of contact.**

**Supplementary questions: Who were you thinking of as non-teaching staff? Is there an average role or job profile in this group?**

**Non-teaching staff: pastoral team, lunchtime assistants, and admin staff. The admin staff informed the average role in this instance. There was very strict adherence to bubbles so this was quite a big difference for this group in terms of contact.**

Prompts: what did you imagine as the job description/daily routine of those who perhaps represented the 5^th^ and 95^th^ centile of your answers

What did you take into account when your provided the new normal values for this group?

**See comments above around children in terms of thinking through uncertainty and adding that to the 5^th^ and 95^th^ centile typically as well as mainly using variance from day to day and within group to pin the behaviour.**

8. Based on experience how did you visualise poor weather affecting contacts?

**Yes, weather is taken into account in answers. It is not the literal weather either which was very good but an imagined typical weather pattern and the consequences for that in terms of wet weather. This increases the number of daily contacts in any one bubble.**

9. Have you subsequently had to deal with children with COVID or thought through some more how to cope with that?

**Had one instance of a child taking a test to shift from Keyworker to year bubble but then also remembered a negative test. In this instance they informed every child in bubble and the teacher and everyone was giving the option of attending or not after that. Only 2 children stopped attending, the test was negative and all resumed. Very happy with protocol but September will be a big challenge.**

[prompts: have you a protocol for contacting other children in their bubble, or for dealing with children or teachers who contract COVID outside of school?}

10. Only if Y to 9 – what happened? Did the reality match the protocol?

**Interview 4.**

**Notes: this is a big school. The new bubble size in September will be 90, current bubble maximum is 10. Year 6 and the Keyworkers came back but Nursery, R and Year 1 worked virtually for reasons described below.**

Question 1. *Most of the questions were in two parts and dealt with pre-COVID and COVID behaviour.*

*The first part usually asked about the behaviour of a typical individual in any year group, and the second was more often thinking about the range across a group.? Can you tell me about how you came about the answer for a typical child.*

(prompts, what is a typical child – is it constructed across year group or school, or by imagining *yourself in a classroom).*

**In many ways imagining what a typical child might be was one of the hardest components of this exercise, as every child is so different, and different day to day. So in this instance thought of a typical child as a typical one behaviourally. So then, in thinking about the variance of the typical child, to some degree the distinction between the two parts of the question were quite difficult to make – was less sure about the difference after rationalising what a ‘typical’ child was.**

**Started out by thinking about each group and running through questions like, what does their classroom look like how many in the class (30 in normal times for this school) how are the seats arranged? Once in class how many times would they go up to each other and up to a teacher? Kids are more fluid and can move around a lot – and then if you thought through something like carpet time for example they are in close proximity to a lot of kids at that point. So thought about the routine the minute-by-minute behaviour and then extrapolated to the number of hours they would be in each situation (in class, in playground and at dinner).**

**The harder to estimate was the pre-covid – it was much harder so many more contacts and things to think about – and daily and difference driven by characters. So it felt much more like normal times was something of a guess- that is how it felt like even though carefully thought through the range of scenarios and possibilities.**

**For the 5^th^ and 95^th^ centile basically thought of those as the minimum and maximum relative to the question – thought through carefully and then added (or reduced) the number to provide some reflection of uncertainty. The middle value was the one that was calculated for the question.**

**The highest value was really hard – because it had to include a normal day, children inside and having free play in a more enclosed space (rainy days), then you also have to account that not only do individual children’s vary between one another but children’s moods vary day to day and that will also vary how they interact with both adults and children.**

**An important thing to reflect on is what will the COVID figure look like now – in September when the bubbles are expanded. The figures used for September will inevitably be much closer to those used in normal times.**

**So what were you thinking about with the children when you were thinking about how that varied?**

**See above**

(prompt: so for example did you think there was an even distribution between the different ways that children behaved – so for example some prefer to play in largish groups together, some form strong pairs, some tend to seek out adult company for reassurance at differing times?)

Question 2. *Related to this (if unanswered by question 1 discussion) when thinking about your 95^th^ or 50^th^ centile levels of contact did you tend to think these were governed by children having larger group work in class or in playground or lots of relatively short-lived single contacts? Were you factoring in behaviour at breaks etc?*

*If yes, can you just briefly run through how children are managed during breaks.*

**See above**

Question 3: so do you broadly think your uncertainty reflected uncertainty in the number of contacts for each question or a wide range in likely behaviours between children and teachers, or on a day to day basis?

**See above. Wide range in behaviours and typical days. With uncertainty thought about maximum and minimum then added in more for different scenarios (eg wet days) then also uncertainty.**

Question 3: Can you talk a little bit about your rationale in moving from the answers you gave for normal times to thinking about new normal.

[what do you think the predominant control was on the reduction in contacts).

**See comments above about difference – did work through as mental model of both days – independently – so from arrival through to all the things likely to happen in a day, or a range of days. Often it’s the transitional points in a day where there is lots of contact. As an example Year 6 might have a really low contact day when separated out and being tested or a really high contact one if their focus is on drama. So used maximums and minimums to help think through the range of scenarios at each time.**

Question 4: when thinking about ‘contacts’ between the children in your school what differences do you perceive between Year 6 and Year 1 children in terms of how they typically conduct work in the classroom and how they typically play during break time and during PE?

4b. Is this a gradual change across the years, or is there a moment when things alter more perceptibly?

**This school did not bring back Nursery, Reception or Year 1 children. Both the keyworker and Year 6 children were in school. The keyworker bubbles did have some examples of younger children however. The assistant head teacher is an Early Years specialist and thought overall asking to comply with social distancing rules would make it very difficult to have true play-based learning and would thus have a negative impact on the children’s wellbeing and development.**

**To some extent there is still some play-based learning in Year 6. Year 6 children are after all still kids so they might have contacts through that. It is important for them to play and to be properly with their friends. But they can understand and rationalise why they are being asked to be socially distant more. To be honest it was also difficult for adults in the school – to genuinely stay 2 m away from one another.**

**For the very young though asking them not to have those attachments in school could be damaging because it’s hard for them to understand why this is happening. After so long away there would inevitably be a need for some settling – tears on leaving of care-givers and this might need contact and we were not allowed to do that. So, on balance we decided we did not want to risk that trauma. We have to accept when we do bring them back that the social distancing will not happen between kids and to focus on good hygiene and trying to protect the adults as much as we can.**

**Another example is the quality of handwashing – its much lower - they can’t pay attention to wash for long enough to remove any virus and kids of that age like to touch everything and they are sneezing and coughing too (even pre-COVID they are taught little rhymes for handwashing and to remind them to sneeze into their elbow). But its important to recognise they are learning all these things, and the point of learning is to make mistakes and for that to visibly be OK for the children.**

**Also reckoned that younger children outside would have more contact and also a lot more touching of equipment outside which is inevitably harder to keep truly clean. Naturally these kids will have a lot of contacts.**

**The change between years is probably organic and happens over time but there is also a change in classroom when they get their own desk and move to separate desk (also giving them their own objects in class that belong to them – rulers etc). In this school that happens in Year 2. Before that the kids are fluid, mobile and moving within the classroom environment.**

5. Did you apply similar rationales in thinking through the questions about teacher contacts?

[to clarify – was the job profile of the person who informed your 50^th^ Centile answer, and that of your 5^th^ centile different for example}

**Yes. Teacher’s was also tricky. Normally this is a very collaborative school and so the teaching staff move about and pop in and out of each other’s classrooms and help one another and have a lot of contact. With COVID we really had to stay in bubbles and the teachers found that very hard – they needed support from one another. The biggest difference of all was which children they were in front of. Younger years also operate in bigger teams so there is more opportunity for adult-adult contact as well as a lot more child-adult contact.**

Supplementary questions: Who were you thinking of as non-teaching staff? Is there an average role or job profile in this group?

**Non teaching staff = senior staff (normally would teach but could not because of cross-bubble contamination); office staff (usually would have lots of contact and now remote) and the cleaning staff (now on site during the day too). There is a big difference for this group and in many ways this is the easiest thing to manage down in terms of contact.**

**Prompts: what did you imagine as the job description/daily routine of those who perhaps represented the 5^th^ and 95^th^ centile of your answers**

**Started by thinking about who the min and max contact days might look like and then took it from there. For some on a low contact day they might never leave their desk in a single room.**

What did you take into account when you provided the new normal values for this group?

**See above.**

8. Based on experience how did you visualise poor weather affecting contacts?

**Yes, there were a couple of days of quite poor weather – this was incorporated see earlier answer. More contacts induced by being in a smaller space for free play.**

9. Have you subsequently had to deal with children with COVID or thought through some more how to cope with that?

**Yes, a child had a high temperature over the course of a day and the school used their protocol. The child was kept in a safe space until parents came to pick them up – take them home and have a test. After this the area was deep cleaned. All bubble parents were immediately informed and had the option to collect immediately or at the end of the day and all children were kept at home until the test came back (it was negative). Parents generally but not universally happy with this.**

**Protocol was followed and it worked well – of course it will be different in September but it helps them think through how this is going to look with much larger ‘bubbles’ to deal with. The experience will be helpful to planning. Helps us think through the process of deep cleaning etc.**

**For some ages of children it’s hard to minimise the contact so what we have to do is really maximise how we practice hygiene on everyone’s behalf.**

10. Only if Y to 9 – what happened? Did the reality match the protocol?

**See above.**

**Interview 5.**

**Notes: This is a teacher from a large split site Junior and infant school. She is head of both but answered the questions from the perspective of the infant school only (R,1,2). This is another school that has Freeflow for the reception class (no fixed classroom). There are about 135 children per year group. They did not bring back the nursery children as the feeling was that they could not mitigate adequately and guarantee safety. This is a school in an area currently experiencing an acceleration in cases. The school has a very high level of parents with English as a second language and in vulnerable BAME groups, so a particular challenge for them has been communication. They have translated many things into Urdu and Punjabi and Romanian and are also working on a strategy with that for September. Largest bubbles here were up to 10 but usually 8 (340 children are in infant school usually). Small bubbles facilitate by relatively few kids who came back.**

**Normally they have 93 children in Reception and just 15 coming in. Many parents opted to keep their children at home.**

**Long discussion about variance and also normal with lots of detail about thinking.**

Introduction – explain who are – purpose of interview is to understand a little bit more about the thought processes and mental models that informed the numerical answers given. This will help the research team to interpret the results that they have obtained from the study in terms of views about contact between children and adults in the School setting.

Question 1. *Most of the questions were in two parts and dealt with preCOVID and COVID behaviour.*

**What I did was begin by thinking through the typical day in the life of a child – not a SEN child – but a middle of the road one who had a fairly typical routine day and would stick to that for the whole day. So went through day mentally in head arrival, playtime, morning.**

**A big thing for us in normal times is that we have early years FREEFLOW (R and nursery). They can choose to go into any one of a number of places/classes and choose what to do. They have a passport and we stamp that to keep track of the types of activities the child is doing. They come together again for snack time and playtime. So a child in this context could have potentially a really high number of contacts. They would be with 4 or 5 children for 5 to 10 minutes and then change and be with a completely different set of kids. There is a quadrangle play area and then teachers around that in each room. These activities could be maths/art/science or role playing etc. Worried a lot that her answers would look funny because this is a LOT Of contacts for these year groups. The kids ‘flit’ about.**

**In this sense these children have less contacts at playtime because they run around more (intimated she had thought a lot about the 5 minute rule in thinking this through).**

*The first part usually asked about the behaviour of a typical individual in any year group, and the second was more often thinking about the range across a group.? Can you tell me about how you came about the answer for a typical child*

(prompts, what is a typical child – is it constructed across year group or school, or by imagining *yourself in a classroom).*

**So what were you thinking about with the children when you were thinking about how that varied?**

**The variance here she thought was largely controlled by the child’s behaviour – so 95^th^ is the kind of child who runs around a lot and has lots of swopping of things and opinions so can see a lot of children. The 5^th^ is the more withdrawn child who perhaps has only one or two closer contacts.**

**In thinking through the cohort question they have a special complex autism unit (children do spend time in the larger classrooms too) and then also a lot of children generally with Special Educational Needs (SEN). In thinking the cohort through these children come into the factor more. Thinking of the extreme child who needs more support and also thus has a lot more contact with adults as well as children. They are in the mainstream classes but some of these kids are pre-verbal with an equivalent learning age of 8-20 months.**

(prompt: so for example did you think there was an even distribution between the different ways that children behaved – so for example some prefer to play in largish groups together, some form strong pairs, some tend to seek out adult company for reassurance at differing times?)

Question 2. *Related to this (if unanswered by question 1 discussion) when thinking about your 95^th^ or 50^th^ centile levels of contact did you tend to think these were governed by children having larger group work in class or in playground or lots of relatively short-lived single contacts? Were you factoring in behaviour at breaks etc?*

**If yes, can you just briefly run through how children are managed during breaks.**

**Answered above but also commented they were really strict now in COVID times with the bubble. They ensured both teachers and children having no contact across the bubbles. She did tend to think that the variance in children’s behaviour was a bigger control on variation than day to day variation in routine, particularly for COVID times.**

Question 3: so do you broadly think your uncertainty reflected uncertainty in the number of contacts for each question or a wide range in likely behaviours between children and teachers, or on a day to day basis?

**Uncertainty was factored into this as well – articulated this more in the context of the teachers but had done it for the most. Thought in terms of the total distribution then asked herself what is the very least number here, what is the very most – how many is that – so then what would the 5^th^ and 95^th^ centile looks like. The uncertainty was factored into her imagined distribution.**

Question 3: Can you talk a little bit about your rationale in moving from the answers you gave for normal times to thinking about new normal.

**Walked through these each in turn. Was very aware how large she felt her numbers for normal times. But we had a lot of children at home in June. They were worried parents. Because of the language issues it took a while for seriousness to get through but then there has been a lot of concern since.**

[what do you think the predominant control was on the reduction in contacts).

**Strictness of the bubble and the loss of freeflow, plus v. few children in.**

Question 4: when thinking about ‘contacts’ between the children in your school what differences do you perceive between Year 6 and Year 1 children in terms of how they typically conduct work in the classroom and how they typically play during break time and during PE?

4b. Is this a gradual change across the years, or is there a moment when things alter more perceptibly?

**The Freeflow is massive. Year 1 and 2 they are in single classes in tables in groups of 8. But the older children do less side by side play and interact and chat more and speak with each other. In their school children’s social skills are poor on entry so this plays into them having less contact in their own time.**

**This changes at different rates for different children.**

5. Did you apply similar rationales in thinking through the questions about teacher contacts?

[to clarify – was the job profile of the person who informed your 50^th^ Centile answer, and that of your 5^th^ centile different for example}

**Yes. Particularly in thinking through the difference between a teacher who works with Freeflow and one who does not. And also thinking about the range of daily interventions needed. Teacher-teacher contact is also a big deal there was a LOT of that and now it’s very strictly controlled. There is however no strong distinction between TAs and teachers.**

Supplementary questions. Who were you thinking of as non-teaching staff? Is there an average role or job profile in this group?

Non-teaching staff. Office, site staff, dinner ladies (not in at all in COVID times). Business manager. They have some variance and pop in and out and would be around fixing stuff.

**New normal = very little contact. No contact with the children but when you think about it many of these staff had contact less than 5 minutes too**.

Prompts: what did you imagine as the job description/daily routine of those who perhaps represented the 5^th^ and 95^th^ centile of your answers

**The typical person was the secretaries. The business manager = super low. Site staff and kitchen = high.**

What did you take into account when you provided the new normal values for this group?

8. Based on experience how did you visualise poor weather affecting contacts?

Not really – because activities are broadly the same did not think the bubble would interact more or less.

9. Have you subsequently had to deal with children with COVID or thought through some more how to cope with that?

**They had an incident with a child presenting symptoms but mother insisting hay fever and mother working at place with known outbreak. So sent home and tested negative. The protocol worked well in this school with high levels of anxiety, coupled with language issue, they would wait for a positive test. They also have a lot of BAME staff so have to think carefully about their protection too. This revealed that their system was quite well thought through.**

**They are really thinking hard about September now. They phoned each parent individually to explain what is happening and are using translated information. The real problem they have is the symptoms: kids cough all the time and the parents use the term fever as a generic word for being ill, particularly when they have more limited English skills so this means they are trying to explain they will have to be specific about fever because there are consequences for each bubble each time.**

**Pre-existing germ policy: well been trying to get the kids into a routine with this since Feb. there are tissues everywhere and they have a good trick with handwashing whether are lined up and given a squirt of soap and then a pause of 20s to do the rubbing before they get hands rinsed. Its quite cool and they sing songs and the kids like it and we make sure they are doing it for the right time.**

[prompts: have you a protocol for contacting other children in their bubble, or for dealing with children or teachers who contract COVID outside of school?}

10. Only if Y to 9 – what happened? Did the reality match the protocol?

**Do you think your answer to elicitation will match closely in September.**

**Somewhere in between – I suspect closer to the new normal because we will not be doing freeflow and really fix their routine. Their bubble will be 30 children indoors going up to the year group (90) outdoors. We are also really on top of adult-adult interaction – that will remain much much lower. That, is the game changer I think.**

**Interview 6.**

**Notes: large school with 640 pupils, 3 forms in each year about 30 pupils in class. Bubbles of 12 with 2 adults. (about 30 children in vulnerable and keyworker bubbles, most of those vulnerable children). Quite a few kids with SEN. About 75% of Year 6 came back but only about 50% of R and Y1.**

**Found elicitation challenging but very happy to do it. Looked up what 95% and 5% centiles were to answer some of the questions.**

**Interview Protocol**

Introduction – explain who we are – purpose of interview is to understand a little bit more about the thought processes and mental models that informed the numerical answers given. This will help the research team to interpret the results that they have obtained from the study in terms of views about contact between children and adults in the School setting.

Question 1. *Most of the questions were in two parts and dealt with preCOVID and COVID behaviour.*

*The first part usually asked about the behaviour of a typical individual in any year group, and the second was more often thinking about the range across a group.? Can you tell me about how you came about the answer for a typical child*

(prompts, what is a typical child – is it constructed across year group or school, or by imagining *yourself in a classroom).*

**A typical child (that’s a tricky question!). A typical child would be one who comes into school every day and then works in the SET classroom environment (I tried a few more times with this but detail is in the description of the school day I think). The classes in this school are set from Year 2 onwards. This means they have three possible classrooms they go into for some core lessons. So this needed to be thought about. So thought about how many children in the classroom at any one time progressing through the school day and then how many in the classroom and rotations with set classes (set here means they are streamed according to stage of progress or level they are working at). In every week there are assemblies and then there are many more kids. Also factored in lunchtimes and breaks.**

**Working this out for pre-COVID times was much harder, and also thinking through the younger children.**

**Further prompts for me to get a sense of what went into variance.**

**Do you have a big school with a lot of outside space? (and then did you increase the amount of time for outside work and factor that in?): Very fortunate to have a massive field, wooded area (they are a FOREST school) and also a separate football pitch and a garden. In COVID times they have 5 lessons a day and for those some were outside meaning they were outside for at least 1 or 2 hours a day of that time (lesson time). There was very little rain so the kids always had that time.**

**Do you have variance in behaviour in the school: We have a high level of SEN kids and so they would usually require 1 to 1 time (did not get to bottom of whether this had changed). There was actually a big problem early on with supporting these children as we also had a high level of adults who were more vulnerable and thus shielding on the staff.**

**Did you think about behaviour differences in the % that you offered. What governed your 95 % answer. Its quite worrying really when you thought through how many contacts a 95% centile child would have.**

**And 5%: that would be certain children who are very shy or who perhaps prefer to spend more time on their own.**

**Do you think there is more homogeneity of experience for all these children in COVID times: Yes, absolutely.**

***So what were you thinking about with the children when you were thinking about how that varied?***

**See above and prompts**

(prompt: so for example did you think there was an even distribution between the different ways that children behaved – so for example some prefer to play in largish groups together, some form strong pairs, some tend to seek out adult company for reassurance at differing times?)

Question 2. *Related to this (if unanswered by question 1 discussion) when thinking about your 95^th^ or 50^th^ centile levels of contact did you tend to think these were governed by children having larger group work in class or in playground or lots of relatively short-lived single contacts? Were you factoring in behaviour at breaks etc?*

*If yes, can you just briefly run through how children are managed during breaks.*

**See above and prompts**

Question 3: so do you broadly think your uncertainty reflected uncertainty in the number of contacts for each question or a wide range in likely behaviours between children and teachers, or on a day to day basis?

**Could not find a way to frame this question so as not to create further confusion**

Question 3: Can you talk a little bit about your rationale in moving from the answers you gave for normal times to thinking about new normal.

[what do you think the predominant control was on the reduction in contacts).

**I moved between the two days mentally and answered the questions. Actually as I answered these questions I did all the ‘normal’ questions first and then came back to them for COVID times. The key thing in moving between the two is that the staff had to be on board with all of the changes to make sure they were implemented strictly. We kept very strictly to the bubbles, which was hard for the teachers.**

Question 4: when thinking about ‘contacts’ between the children in your school what differences do you perceive between Year 6 and Year 1 children in terms of how they typically conduct work in the classroom and how they typically play during break time and during PE?

4b. Is this a gradual change across the years, or is there a moment when things alter more perceptibly?

**There is a big difference between the two – again the reception children here have FREEFLOW (moving freely between classrooms in normal times and play led learning). There is also a variance in class with desk arrangement and then also carpet time. Actually the younger kids are in smaller sub-groups (so e.g. carpet time in normal times is with 10-15 at a time. Year 1 is also not set so the Year 6 have more movement during class time because of the setting.**

**How does these changes work in the playground? Lower down the school the kids are much more touchy-feely with each other and affectionate. Less so later on. The differences between the years are gradual though and for some children they remain like that even in Year 6 (further prompt: did that feed into your variance then? – Yes).**

5. Did you apply similar rationales in thinking through the questions about teacher contacts?

**Yes. Similar procedure in everyday – thinking through a typical day with sets and then of course thinking about the fact they get half a day preparation time every week which is when you have no contacts.**

***Prompt: so what shaped your thinking about 5^th^ and 95^th^ centile*- it was much more the daily variation and to some extent that was shaped by likely variation in adult contact. Also said that TAs and teaching staff have broadly similar levels of contact.**

[to clarify – was the job profile of the person who informed your 50^th^ Centile answer, and that of your 5^th^ centile different for example}

Supplementary questions. Who were you thinking of as non-teaching staff? Is there an average role or job profile in this group?

**Non-teaching staff are office staff, lunchtime supervisors, dinner ladies and on site supervisors. The typical version of that is those who were coming in every day and probably the office staff – who have gone to having almost no child or adult contact.**

**The children have dinner in their bubbles which is pre-ordered and delivered to the classroom in a box. At lunchtime the children had no opportunity to interact outside their bubbles.**

Prompts: what did you imagine as the job description/daily routine of those who perhaps represented the 5^th^ and 95^th^ centile of your answers

What did you take into account when you provided the new normal values for this group?

8. Based on experience how did you visualise poor weather affecting contacts?

**It would not really affect things as we were so strict with the bubble and that will remain within their 30 children bubbles. (implicit in this is that contact was larger within the bubble). I probed on this and she said that the children were initially very good but as time went on they started to forget about it within the bubbles and got lax. She also remarked they would be good at keeping bubbles separate but as soon as they saw each other at the school gates there would be a huddle.**

9. Have you subsequently had to deal with children with COVID or thought through some more how to cope with that?

[prompts: have you a protocol for contacting other children in their bubble, or for dealing with children or teachers who contract COVID outside of school?}

**No.**

**Asked some questions about protocol: they have a separate first aid room and kid with symptoms immediately out and looked after and protocol to inform bubble if case confirmed.**

10. Only if Y to 9 – what happened? Did the reality match the protocol?

**Thought protocol would work well.**

**To close I asked about which answers might apply more in September. She responded that probably the answers would be in between as they will stick with the strict 30 bubble and not have freeflow but obviously the bubbles are larger.**

**Section 3. Non-attributable verbatim responses to risk mitigation questions**

*[1a] Please describe your strategy to reduce close contacts between pupils (about 50 words). You might want to discriminate between Cohort 1 and 2 above.*

- Social distancing (marked out on floors using tape). Large rugs used in cohort 1 with little ticks on the ‘bugs’ where they sit, Green ticks used on tables in classrooms showing where pupils to sit in cohort 2, Signs all around the school, Playground marked out
- Reduced groups to 10 children to fit into a class with social distancing. Using Hall for key children. Removing furniture to ensure safe social distancing. Visual aids to remind children of what 2 metres actually is. Adult supervision to reinforce social distancing. Individual desks and equipment/resources. Use of outside space for older children.
- Children are in bubbles of up to 15 in separate classrooms with separate playground spaces and allocated toilets. Children are all part-time so that we reduce the number of people on site at any one time and ensure that all staff are only allocated to one bubble.
- Teaching staff are generally assigned to a single bubble where possible but in some instances teaching staff teach across two bubbles. Children are taught in groups of less than 15 in separate classrooms. Use of staggered home time and arrival times different from Cohort 1. Each group of children to have separate play area, toilets, canteen space, and routes to the play area. Each pupil to have their own stationery in class. All books and materials kept on tables to reduce children moving around. Practice of new routines (e.g. moving around school). Discussion with children about more challenging areas (e.g. playing in the playground)
- In cohort 1 we have 2 bubbles 1 of 15 across 2 rooms 1 of 7 in 1 room they all are outside as much as possible. In cohort 2 we have 4 bubbles the maximum size is 15. most are across 2 rooms or have moved to classrooms with greater area.
- Answers:
  - Bubbles of 15 that do not mix
  - Zoned outdoor areas
  - Spaced out desks and positions in class
  - Resources left in school
  - Resources sterilized daily
  - Lunches in bubble groups
  - Increased outdoor learning time
  - Distancing markers around the school
  - Staggered starts and pick up times
- Answers:
  - Cohort 1 – More outdoor play, space between desks, some desks in Reception (none before), lining up & moving around school with spaces, gaps on carpet, drop off and pick up staggered every 5 minutes, separate toilet cubicles
  - Cohort 2 – Spaces between desks, work at desks, spaced lining up and movement around school with spaces, 1 toilet cubicle per bubble, no cross bubble toilet visits, one way system around outside of school and in corridor, staggered drop-off, pick-up, more outdoor learning.
- We have adopted a mixed model with classroom of a max of between 12 and 15 children, in y1 upwards they all have their own desk but they are not spread out to a strict 2m distance about, as our rooms are small, what we have done is made sure everyone is facing the same way and no one sat facing another person, and that there is a gap between every desk so each child is always at least 1 m and nearly 2 m away from each other when seated. We use outdoor learning as much as possible so all children are outdoors for at least 30% of their day. In year R, we have gone for outdoor learning as the norm, with children interacting as usual in their pod of 12-14 children but doing the majority (80%) of their learning outdoors.

Answers:

- - Cohort 1: In nursery we have three rooms 0-18 months, 18 months -36months and age 3-4. Each group is in separate rooms in a shared building and in each room there is a physical divide to separate the key groups. Staff identified for nappy changing and trained in increased cleaning and managing the separated space for intimate care. Key groups are the maximum size of three for 0-36 months, two groups can mix. Age 3-4 groups are separated. EYTS responsible for planning but two groups delivered by HLTA and TLA no more than 6 in each group. Separate building from all other key groups. Reception teacher and TLA running two groups over two separated rooms. In Y1 children from one class split between two rooms, with identified working areas fulfilling 2m distance. TLA and teacher In all groups children in the same year group play at the same time but in identified areas.
  - Cohort 2: These students have been allocated working areas that maintained a 2m distance and students have been in bubbles of between 8 and 12. One teacher has done all of the planning and delivered in one room whilst it is screened to the adjoining room and supported by a qualified TLA or another teacher in most cases. Children arrive at school at staggered times, routes are detailed to avoid other groups as far as possible and where there is a passing it is brief and corridors are divided. Year 6 are working in our senior school to enable us to allocate every year group a dedicated break area. All children are self sufficient including bringing packed lunches. We use a specialist teaching approach and specialist teachers are delivering ‘live’ digitally so they don’t enter any bubble.
- Reception children have access to setting almost as normal with soft furnishings removed. They sit spaced apart on the carpet and are directed to small group work of 4 pupils with an adut. They also have free-choice times of activities set out by the staff. Provision is adult led not child initiated. Year 1 and Year 6 are at individual desks – 2m apart with individual trays of resources. They mix at playtime and lunch times in their small groups in a large playground. Lunch is in classrooms all groups – 1 child per table – all packed lunches (whether from home or school). All groups in just 2 days a week.
- Answers:
  - Cohort 1 - nursery and reception are sharing the same room and Year 1 are in their own room, where tables have been removed and the children are either inside or outside at certain times of the day, soft furnishing and half of the tables and chairs have been removed, toys are rotated on a weekly basis with certain toys only used on certain days.
  - Cohort 2 - as above but with year groups in separate rooms.
- N/A
- Answers:
  - Cohort 1 only attending if key worker or identified as a vulnerable family.
  - Cohort 2 children also only key worker or vulnerable families for Y2-Y5.
  - Y6 children have the option to return
  - All children have been organised into bubble groups, with a maximum of 10 children in each bubble. Bubble groups to remain separate at all times and not mix with each other.
  - Desks within bubble groups to be socially distanced, each child to have their own set of resources.
  - Staggered entry, pick up and lunch times to ensure bubble groups do not mix
  - One way system in place for parents in the playground
  - Playground areas divided into zones for different bubble groups.
- Answers:
  - Children remain in their own decluttered classes to maintain the bubble
  - Children have their own space and/or desk separated by 2 metres from others
  - School field is divided so children do not mix bubbles during break
  - Entry and exit to classes is directly from outside (children do not use the corridors in the except to go to the toilet)
  - Toilet areas used by one child at a time
  - Children spend over 1 ½ hours each day outside
  - No school clubs or activities that would bring children into close contact
- Limiting numbers in classes (half the class in at any one time with a max of 12 in any one class) – 1 group in Monday and Tuesday, school closed Wednesday for a deep clean, the second group in Thursday and Friday. Each group in school is a bubble and stays only with their teacher for the day (including break and lunch) and uses separate toilets. Each group shouldn’t be mixing with other groups. Breaks and lunches staggered for each group in school and lunch eaten in the classrooms to reduce contact in dining hall. Lunches delivered to classrooms in takeaway cartons so it is all fully disposable. Teacher fills water bottles from a jug so lots of pupil hands not on taps. Water fountain not in use. Pupils not to bring anything from school to home in and vice versa (apart from lunch boxes). Outdoor learning time time tabled so bubbles don’t mix outside. Cohort 2 using minimal paper for work – work on computers in school for teachers to mark to reduce contact between teacher and pupil.
- Cohort 2 - Children are seated at desks that all face in the same direction (towards front of classroom), 2m apart during all lessons. Children do not change desks at all for lessons. Classes move around the school (e.g. to go out to break) at different times to avoid contact in narrow corridors. Children in school at present (R, 1, 6, plus keyworkers’) are spread across rooms usually occupied by other classes to maintain this distance inside. Cohort 1 – slight variation in that children don’t sit at tables for as long, but are still encouraged to keep the 2m distancing in the classroom. Note – this is a very small school, with all class sizes significantly below national averages.
- N/A
- Answers:
  - We are inviting children in in ‘bubbles’ of no more than 10. They will be in one classroom with 1 teacher, 1 TA and will have their own lunchtime supervisor – so closed bubbles, with under 10 children and 3 adults.
  - As an infant only school, we only have to welcome Nursery, reception and Year 1. Our trust have risk assessed and NOT included nursery children – they could not mitigate risks. Hence there are no cohort 2 children involved (if you wish I can repeat this for my Junior school who only have Y6 in – please let me know)
  - Staggered drop offs, 1 adult per child on site, staggered playtimes (using different areas and equipment), staggered lunchtimes (Separate areas, tables and equipment – and hot or cold ‘grab bags’ no cutlery and all disposable.
  - Tables spaced 2 m apart, reception children encouraged to ‘play’ in small groups of no more than 3 in separate areas of the class, and work individually at well resourced tables.
  - Y1- separate tables, own resources.
  - We also have a resource base for children with complex autism – they are in part time, to allow small bubbles, individual resources, no sensory rooms, or sift play to be accessed. Staff can wear PPE if they wish as they have to deal with personal care of these children many of whom are pre verbal.
- There has been a one way system implemented inside the school building as well as the playground. The children are in ‘bubbles’ of no more than 12 children and they do not mix with other ‘bubbles’. The children all have lunch at different times with a 15 minute slot for each group. The outdoor time has been carefull timetabled so only one ‘bubble’ is in one place at any given time. There are markings on the floor so the children know where to stand.
- Answers:
  - **Cohort 1;**

Nursery x 1 bubble (3 children)

Reception x 1 bubble (3 children)

Yr 1 x 2 bubbles (3 and 6 children)

Key worker bubbles x 2 also include R and Yr 1 children

**Cohort 2;**

Yr 6 x 3 bubbles (6, 6, and 8 children)

Key worker bubble x 1 of Yrs 3, 4 and 5

- - Bubbles are spaced out across 4 buildings with their own entrance and exits. They are allocated outside space to play. Lunches are delivered to classrooms. Each bubble has two members of staff who take it in turns for lunch and breaks.
- Pupils have been spilt into max bubbles of 12. They have 2 adults with them and they stay in the bubble. Only yaer6 have been invited in and for 2 half days. No other pupils have been invited as yet so no cohort1.
- Nursery - we have recognised that it is impossible to prevent close contact of nursery age children as by nature they play closely and physically and staff need to make contact with them during the day to maintain their welfare and safety - however the staff have undertaken a stringent regime of cleaning resources as well as the play area during the day and are maintaining about 90% time outdoors. R/1/6 spaced desks, minimal contact during teaching sessions, spot markers for lining up, one way system through school, reduced number of children using toilet facilities at a time to reduce crowding. Doors and windows propped open. Also an extensive part of the day is outdoor learning although with the younger children, this is often when close contact happens. There are staggered playtimes and lunchtimes as well as restricted use of playground equipment, climbarounds etc.
- As a cohort2 school we have taken a very cautious approach to the reintegration of pupils into school. In order to observe the 2m social distancing requirement we have organised year 6 into bubbles of 5 pupils. We are currently only allowing 2 bubbles of 5in at any one time and they are located in separate classrooms. The classrooms have safe zones taped out on the floor and social distancing tape is used when pupils are outside of the building

*[18] Describe briefly the cleaning regime in your school using disinfectant (maximum 50 words)*

- There is a specially designed rota for cleaning at this time. Classrooms and workspaces are disinfected prior to the school day, twice during the school day and then again at the end of the school day. Cleaning staff disinfect areas other than classrooms prior to the start of the school day.
- We have one cleaner who cleans all areas for 2 hours at the end of the day. We also have now engaged a new cleaner to clean all through the day (6 hours) specifically concentrating on toilets and high use items (computer suite) and touch points. All areas are visited a number of times a day on a rota and she is re-tasked at the request of other staff requiring specific areas cleaned.
- Desk and communal areas in classrooms that have been touched cleaned down at the end of every session – 4 times a day. Outside play equipment cleaned down between different groups. Four times a day, high-traffic areas cleaned down. Disinfectant spray in toilets, and staffroom and communal areas with disposable cloths for people to us.
- Clearers wear PPE, and PPE is changed when moving from one ‘bubble’ to another.

1- wash with soap and water

2- wash with disinfectant

All PPE and cloths are double bagged and stored for 72 hours before putting in bins. Door wedged open and toilets are steam cleaned twice weekly, and cleaned as normal at intervals during the day (each class has a set cubicle and sink to use). Each ‘bubble’ has own PPE and cleaning resources to use during the day- tables and surfaces regularly cleaned. Cloths double bagged.

- The cleaner disinfects all surfaces every morning, we then disinfect the taps, toilet flush etc. every play time and lunch time, desks and chairs every lunchtime and then we do the same at the end of the day. We also disinfect laptops every time they are used, even though they have one each, and pencils rubbers etc. once a day though they have their own.
- School is cleaned before the children come in and after they have left on an evening, where the floors are mopped; the toilets are cleaned with disinfectant and the bins emptied. In addition the tables in the classrooms and toilets are wiped down during break and again at lunch time with disinfectant wipes and the bins are emptied as well.
- All used areas of the school are cleaned daily in normal circumstances. Under the ‘new normal’ a cleaner remains on site each day to regularly clean the frequently used areas such as toilets and the frequently touched surfaces such as door handles and stair rails. All staff and children must sanitise their hands on entry to school.
- Toilets and doors to toilets cleaned 3 times/day. Desks cleaned during morning break and/or lunch break, Whole school cleaned daily (after school)
- We have significant allergies in some of our children so cannot use disinfectant in communal areas/ toilet/ the classrooms of these children. This is severely hampering our ability to deep clean. Doors kept open to reduce need to disinfect handles. Hot water and soap used in most areas due to allergies to disinfectant
- At the end of each session (e.g. before morning/afternoon break time or lunch), all equipment used in session is wiped with disinfectant wiped. At end of school day, all surfaces are wiped over by children and teachers/TAs. Regular full cleaning takes place after school with increased focus on surfaces, door handles, banisters, etc. with disinfectant. Additional spray sanitisers are placed throughout school. These are used directly after any tissue is used and placed in the bin; whenever adult communal equipment is used (e.g. before and after use of photocopier, laminator, paper cutters, staplers, etc.); also adults use these when they come into contact with items children have been using.
- We deep clean at the end of every day in heavy use areas (10 children plus 2 adults). All resources cleaned during the day. Deep clean across school on Friday.
- Usual clean every day at each site – 2 hours x 4 people = 8 hours regular clean. Twice daily surface clean during the school day of high-contact surfaces e.g. door handles, exit buttons, etc. Deep clean twice a week between bubble groups using the same spaces. Any shared resources are sterilised using Milton’s solution between groups, e.g. in EYFS. Resources are not shared unless they have been sterilised.
- Use of anti-viral cleaners twice a day on hard surfaces, Milton sterilising tablets in solution at end of each day to sanitise hard plastic toys
- Tables, chairs, toys and equipment, toilets, carpets, walls (wiped down) all cleaned before children come in in the morning. Wednesday day and Friday evening, school given a deep clean to ensure whole school is ready for next set of ‘bubbles’. Toilets cleaned 3 x a day when pupils are outside
- Cleaning is performed three times per day.
  - Morning before class: Tables are wiped with disinfectant (80% alcohol). Check done on rooms, toilets and remedial action taken where necessary.
  - Lunchtime: Toilets cleaned, Table tops cleaned
  - After School: rooms cleaned thoroughly include tables, chairs, floors mopped, carpets vacuumed, lobby areas, toilets, handles, stair rails

Each classroom has supply of disinfectant and paper towels to do any extra cleaning. Toys are cleaned by teachers . Computers and keyboards are wiped down by teachers.

- The school cleaners enter the classrooms at the end of the day when they children have left the site. All the tables and door handles are cleaned. Toilets are cleaned. Shared equipment (Nursery/ Reception) is cleaned.
- Each classroom has hand cleaning facility, toilets located nearby. Classrooms are cleaned daily and a deep clean at the end of the week.
- We have employed an additional two daytime cleaners who are cleaning door handles, bathrooms etc. The cleaners are completing additional deep cleans to classrooms every day. There is a sanitation station in every classroom. There are now hand sanitizer dispensers on walls around the school.
- Nursery – very stringent, all toys played with during the day are sterilised with disinfectant everyday. Outdoor toys sprayed with antibacterial spray. Table backs of chairs, computers, doors, handles, sinks, taps, toilets cleaned 5-6 times a day with disinfectant spray and disposable cloths or paper towels. R/1/6 – tables, chairs, door handles, computers wiped down twice a day, lunchtime and at the end of the day. All communal spaces cleaned thoroughly by cleaners after school including toilets.
- Teachers and teaching assistants sterilise equipment at the end of each day, External cleaners clean every day after school, external cleaners do a deep clean once a week when the school is closed
- Every used classroom cleaned every day, Loos cleaner 3x a day
- End of each day each space is deep cleaned by the contractors, all surfaces and floors and doors, cleaner onsite all-day cleaning touch points and emptying lidded bins. Bubble allocated toilets cleaned at regular intervals. Equipment such as toys on rotation and cleaned at the end of each morning and end of day by EYFS staff using soapy water and Milton solution.
- 7-8.30 AM week days 3 cleaners x 2.5 hours doing clean of surfaces, toilets, carpets etc in all classrooms and throughout school; 10-11am 1 Midday supervisor 1 hour cleaning toilets and sinks and touch points in toilets e.g. doors; 12-1 3 Midday supervisors 1 hour as above for toilets plus cleaning down all tables in classrooms, touch points on main doors and office doors and shared surfaces including staff room kitchen area; After school 4pm teachers wipe down keyboards, laptops and tables and shared surfaces in classroom they use

*[19] Describe the policy for parents to drop off and pick up their children each day (50 words maximum).*

- Cohorts given staggered times for drop off and pick up. Parents asked to wait across on the green in social distanced way until children are brought out by class teacher and sent across to parent one by one. At drop off senior member of staff stands at gate giving room for social distancing and children welcomed in and walk immediately round to their classrooms. No visitors or parents on school site. Children arriving by car met from car by staff and they walk straight into school.
- There is a one way system that has been put into place in the playground, parents enter via one gate and leave through another. There is a 20 minute window slot for drop off and pick-ups. During pick up times, a member of SLT stands in the playground with a walkie talkie, they then radio across to different classes so they know who to dismiss (KS2). This is to minimize the teacher contact with other adults.
- Only yr6 re back so they have a drop off time and place. It is and would normally be different to yr1 and reception
- Staggered start and finish times to designated gates. One group of 15 comes in each gate every 10 minutes and parents (one parent only) and children wait on social distancing markers. Parents are not allowed past the gate unless prior permission has been given.
- Staggered entry times and pick up times. One way system for parents entering and exiting school gates. Portable sinks outside, parents and children must wash hands once they enter school grounds
- One way system in place with 2m markers on the ground to impose social distancing. Each child is dropped off at a barrier and child is registered and escorted to playground area. At pick up children wait in their bubbles outside to be collected by parents (singly)
- There is a staggered dropoff, done by family rather than year group, and parents must leave their child at the gate.
- Parents/carers do not enter school site. They arrive outside the school gate and their children line on socially distance markings. Collection is the reverse. A percentage of year 6 pupils come to school on their own.
- Staggered drop off/collection. Parents expected to adhere to social distancing outside school. Children dropped off and met at school entrance (no parents come into school at these times).
- Parents to drop their child at the gate and leave. We are not staggering start times due to the complications with family groups being dropped off that this caused. All pupils who can walk to school alone (most live close) to do so. Pupils to go straight to the fire exit of their class (which is kept open so they don’t need to queue) and not play in the playground. Each class enters and leaves through their fire exit door so there isn’t contact at the front entrance. End of day, family groups leave alphabetically, parents wait in that order at the gate.
- Times staggered by year group and exits and entrances controlled so there is no cross over (physically) of children and parents/carers.
- If possible children walk into the school alone and walk directly to their classrooms or wait on field outside their classroom; Parents bring children round the school (outside) socially distancing and wait until child can enter building; Parents leave via different gate so that they are not meeting others coming in (loop round the school); Each class has a different drop off/pick up time.
- Bubbles are spread aross 4 school buildings. No more than three bubbles share an entrance or exit. Parents are not allowed on site but most stand at 2m markers on the pavement in they need to queue.
- Drop off and pick up is staggered with 15 minutes between each group (maximum of 10 children in one area at one time). Children are dropped off in the playground, they stand on a spot on the ground (2m apart) whilst they wait for their peers to arrive. All children have a temperature check and sanitise their hands on arrival. A one way system is in place for parents to enter and exit the playground. The adult leading the bubble group greet the children.
- 6 groups, dropped off at 5 minute intervals (one additional group for sibling drop off). Only one parent dropping, one-way system, social distancing. Temperature taken before entering.
- We have a one way system for each group and a separate entrance for each group. Parents must come in via one gate and exit via another, leaving their child to come into school on their own. We have an extended drop off time in the morning of 20 minutes as 3 separate gates are used and phase pick up in the evening: 3.00, 3.10 and 3.20 pm. If parents arrive early they are expected to wait while socially distancing on the far side of the road to the school. If gates are open at pick up/drop off, they can come onto playground in a given area and wait while maintain social distancing.
- Parents drop their children at the main entrance and the children walk to their bubble. On collection a walkie talkie is used to alert bubbles which children need to be sent to the main office to be released.
- Staggered starts and end of the day. One way system around the school. Youngest in first and leave first. Oldest arrive last and leave last. Have to be on time. Two entrances into the school – one used for the key worker vulnerable, one used for rest of the school.
- 1 adult per child on site; Each bubble has a different door to enter/ exit. 2m lines painted outside each door for queueing- children enter/ exit 1 at a time, School office closed, communication by text, email or phone- if a parent needs to come in, staff go outside to them and distance. There are 3 staggered drop off and pick up times with a window of 10 minutes for each. One parent is allowed on site at any one time. There are floor guide markers to indicate a one way system and lining up distanced spaces. The teacher receives and dismisses children at the door.
- Staggered times- no bubble mixes, O parents in school
- Staggered, 10 mins separating as minimum depending on year group size. Markings for social distancing.
- Over a period of one hour, the ten pods have different drop off and pick up 15 mins slots to avoid congestion. There is a one way system on the playground and parents wait in the playground beyond the hedge rather than coming right up to the doors. There are lines on the floor to show them where to wait if there is a queue for a pod. They are encouraged to head straight off out of the playground!

*[22] What is your policy if a child or adult staff develops possible COVID19 symptoms (either outside school or during school hours) (50 words maximum)?*

- They will be sent home if they develop the sympotms within school. They will also be removed from the bubble at the time the symptoms develop. We have a first aider on hand the whole time. There is a separate self-isolation room that has been set up for both children and staff if they develop symptoms. If they test positive then their whole bubble will be sent home and need to self-isolate for 14 days.
- Isolate child immediately in school and call staff from office to come and supervise. Pernt called and asked to collect child immediately and have them tested. If test is positive the whole bubble isolates for 14 days and room is deep cleaned and then not used.
- Person is isolated from other people. If a child they are required to be collected as soon as possible by a parent/carer. Adults would be required to go home and arrange for a covid test to be carried out. They would be required to follow the government isolation advice until such a time that the test results return negative. Outside of school hours school to be informed so as to determine level of risk. Again staff to get tested.
- Group with whom they are working will be asked to self-isolate-adults and children. Deep cleaning of classroom affected immediately.
- They go home immediately, other parents in bubble notified, person sent for test, if test positive whole school would shut.
- Sent home to self-isolate until 7 days have passed or have had negative antigen test.
- Mainly following the DFE guidance. If outside school hours, child or staff member must not come in, inform us that seeking testing and inform us of the result. If during school day, isolate that person immediately (under supervision if a child), call parent to collect or send member of staff home. The DFE guidance states that bubbles only close if a positive test is returned – we are going further than that and sending the bubbles home to start self-isolating whilst awaiting the results of the test. This is to reduce the likelihood of transmission whilst waiting for results.
- Outside school: There must stay and home if they develop any symptoms. Contact the school and use NHS 111 service and follow guidelines. Within school: They are brought to an isolation room where they must wait to be picked up by parents or family (staff may go home in own car). They must phone NHS 111 service and follow guidelines
- They must go home and be tested. Not allowed back until the test results arrive and they are clear. If they are not cleraed, stay home. Rest of the group informed. Sent home to be tested.
- Follow local health protection team guidelines (provided by local education authority). Any child arriving at school gate with signs of a temperature is sent home immediately and must remain at home until a negative test result is achieved. Expected to isolate along with household for 8 days (7 days plus day of commencement) at home. If child/adult develops symptoms in school, same procedure applies. Any positive result (confirmed case outside school), the health protection team conduct a risk assessment to determine others potentially at risk at school and school follows their guidance, only closing class/school if advice received to do so. Deep clean undertaken as advised and school continues as ‘normal’ unless advised otherwise. If 2+ cases develop in same group in school, whole group required to isolate and be tested, only returning if test negative. School shut for additional deep clean for day before all other groups return. In all instances, health protection team will follow up on contacts in group, carry out risk assessment and determine whether specific groups or whole school should be sent into isolation or return.
- Person to self isolate, along with any people they have had contact with immediately and get tested. Parents of children the person has been in contact with informed. School closed for deep clean.
- Follow PHE guidelines/flowchart and phone them for advice/guidance; Child taken to area away from class/adults sent home and asked to get tested for COVID; Adult attending child to wear PPE; Child collected by parent ASAP – instruct parent to have child tested
- PPE used (if in school) and sent home – insist on a test. Wait for the test result – if positive, close the bubble and ask staff and pupils to isolate for 14 days. Contact Public Health England
- Parents expected to pick child up. Child is taken to medical room and supervised at 2 m distance by an adult. If adult has to provide close support they must put on PPE – face mask, goggles, apron and gloves. Child is kept in medical room with windows open and door open until parent collects. Child expected to be tested and kept at home until test result in. Parent must inform school of result of test. Once child has left the area they have been in is disinfected – medial room and their desk space. PPE disposed of and double bagged. Staff member must go home and get tested as soon as possible. They are expected to stay home until result of test if negative even if feeling better – expected to inform Headteacher of result as soon as received.
- In school: If a child or staff member develops symptoms then they are taken to the self isolation area until they can be sent home. They will be asked to book a test and remain at home until the results are back. At home: If someone (a relative) has symptoms then they are asked to inform school and book a test. They will isolate until their results come back. If the result is positive then the bubble attached to that child/staff member will have to isolate for 14 days.
- They must isolate in school until they can leave and then must be tested and inform school of the result ASAP. The room where they have been isolating must be deep cleaned immediately ; If the test is positive the whole of their bubble group must isolate for 14 days; School will be closed to all for 2 days for a deep clean.
- Follow DfE guidance. Taken to first aid area, and 1^st^ aider wears appropriate PPE. Phone home to be collected. Test is strongly advised to be taken. Parents informed; Bubble continues – if test results comes back positive – all self-isolate. If negative – carry on and child returns when well.
- Follow PHe guidance. Out of school – get a test remain at home until results are through. If positive test and they have been in school, speak with contract tracer and potentially 14 day isolation for everyone in that bubble. IN SCHOOL – Staff go home, get a test and follow abover guidance; Children- assigned room, alone of possible (if not, staff wear PPE) – windows open, call parents to collect – use assigned toilet if needed. In either case- all symptomatic people asked to get a test, remain at home until result- negative- return, positive stay at home and bubble isolates.
- Send them home and take a test as soon as possible. Keep the bubble open until the results are back. If positive close down the bubble for 14 days.
- Current policy:
  - Children:
    - Child is taken to isolated room
    - Staff wear PPE
    - Toilet for child showing symptoms is not shared by others
    - Parent is called immediately to collect
    - Child is asked to get tested
    - The bubble goes off until the test returns negative or after a 7 day period
  - Adult
    - Wears PPE. goes home and self-isolates for 7 days / is tested
    - The bubble goes off until the test returns negative or after a 7 day period
- PPE and isolation
- Follow the flow chart provided by the local authority. If inside isolate and nominated member of staff using all recommended PPE has the contact. Parents called immediately and testing requested and results to be shared immediately. Deep cleaning of the medical room (isolation space). Symptoms outside, pupil not allowed in school and must go for testing and results shared immediately.
- Call home for immediate pick up, various letters to inform other pod members if there is a positive test, call PHE local contact

**[23] What is your policy if a parent or another relative of a child contracts COVID19?**

- The child would stay at home and self isolate with the family for 14 days
- Children may still attend setting
- That child is to self-isolate for 14 days from when the symptoms first. Other parents in the bubble to be informed so they can make a decision on whether to bring their child in or not.
- We have had advice from the local Health Protection Team of Public Health England on this in the form of a diagram which we will follow.
- Child isolates for 14 days with household as per guidance. No further action unless child is symptomatic and tests positive- then as above
- The child would then need to self-isolate. The school would also need to be informed.
- Parents must keep their child at home to self-isolate for 14 days.
- As above with child sent for test They go home immediately, other parents in bubble notified, person sent for test, if test positive whole school would shut.
- The definition of a contact includes: any child or staff member living in the same household as a confirmed case, or equivalent setting such as boarding school dormitory or other student accommodation; so, we would expect them to inform the school and contact NHS 111 and follow guidelines.
- The child has to isolate for 14 days and must be tested. If they test positive the policy above would apply.
- Policy depends on if the child is living with the individual. If they are then they must not come into school. Child would be expected to get tested before coming back into school having followed government guidance. We would inform all those who the child has had contact with that they have had contact with someone with the virus.
- We would recommend that if they experience symptoms they should not come into school. If the child then tested positive, we would expect everyone they have had contact with to not come into school, get tested and self-isolate until results come in. If they are not, they must not have contact until individual is clear of virus.
- Follow the government guidelines of the 7 day and 14 day isolation period if from the same household
- If someone (a relative) has symptoms then they are asked to inform school and book a test. They will isolate until their results come back. If the result is positive then the bubble attached to that child/staff member will have to isolate for 14 days.
- Child is asked to isolate for 14 days and have the test
- Family to self-isolate for 14 days, the child is not to attend school during this time and will receive online teaching.
- Standard government requirements for self/household isolation and testing – child returns when test negative.
- Child asked to stay at home for appropriate number of days.
- Nothing. If a child has symptoms then be tested
- the family should self-isolate until tested negative. Child can return after 7 days.
- No policy
- As a contact of someone tested self-isolation for 14 days. Don’t require test unless developed symptoms.
- We would ask that that child remains at home for 14 days but would not close the pod

*[24] Other Comments. Please make any remarks about additional risk factors that you think should be considered (maximum 100 words).*

- School has been very slow and very measured. Only 24 yr6 are in out of 120. Bubble size is 12 but only 6 have come for each bubble. Not yet open to other year groups.
- Not able to access cleaning products. Budgetary considerations. Staff not available or reluctant to cover others.
- We think we are quite lucky as all our classrooms have separate outside doors, we have 3 sets of toilets between 4 classrooms and a playground with lots of smaller sections so we each have a section we can use whenever we want.
- In this very small school, only 1 non-teaching/TA employee; probably unlike other schools. Most unlikely to spend significant time with children in this setting. TA duties include supervising lunch, which again might be unusual use of their time. TAs may spend more time with a single child. TA also more likely than class teacher to spend 1-1 time with many more children (e.g. listening to each child in class read) and in more than 1 class, increasing likely closer contact with individual children. SEN children may exhibit more extremes of contact behaviour (from most contact to least contact with others) – this may change depending on situation (e.g. whether in class or during breaks). Equipment – question potential for increased risk due to children touching/sharing school resources (e.g. science equipment, toys/ play resources, stationery); this is not a suggestion that there is an increased risk, rather a question as to whether this is likely to have an effect.
- Staff with family members/partners with underlying conditions have been asked to consider their ability to come into work. Pregnant staff have been told not to come in to work in order to minimise risk of infection as much as possible.
- Uniform – some pupils are not washing their clothes each day so there is increased movement of things between home and school. PE – at the moment we are doing PE outside (although we have an issue with outside space as breaks and lunches are staggered and so most of the day, a class is on break outside!) however this will be an issue as we move into Autumn/ winter. Outdoor learning – as above, although we have tried to implement outdoor learning as a safer way to teach, outside space is a limitation due to the staggering of break and lunch; Contact between teacher and pupil more of an issue with the younger pupils as they cannot work online and so there is paper transmission between teacher and pupil. Challenging pupils refusing to adhere to distancing difficult without parental support
- I’m sure that you’ll have quite a mixture of types of schools involved in the study, all of which will have their own unique set of challenges. For us, there are two things that make our situation different from some schools. 1) we’re quite a big school with three forms of entry in each year group so that means there is the potential for large numbers of contacts on a normal day; 2) we’re split across two sites so if we had a cluster of positive Covid-19 cases at one site, it wouldn’t necessarily mean a cross-transmission to the other site unless parents, siblings or staff had aided that transmission.
- Within school we now have four separate staff rooms to ensure that adults are not all in the same space at the same time.
- Within my school we are mixing a small number of children between bubbles as we have a wrap around provision before and after school, where children are placed before 08:45.At 08:45 the children wash their hands and go to their class bubble. In addition to this, any children still at school after 15:45 are put into and after school bubble until they can be picked up. Finally, we are also having a staff briefing each morning in a small staff room where we cannot social distance (and with no windows open).
- Some children & families (and possibly staff) breach regulations outside. We suspect there is some breaching but cannot definitively confirm. Question 8a – when asking staff to estimate contacts there is a significant difference in the quantity of contacts between adults working in KS1 & KS2. In KS1 there is much more close contact, often in groups.
- Additional risk factor centre around the contacts of children and adults outside of school. In school we have them in bubbles, they may not be with their friends, and outside they may socialise with their friends, so cross contaminating bubbles. Also travelling to different areas where incidences are higher can lead to the pickup of virus and transmission back.
- Questions 10 & 11 are difficult to answer with any degree of accuracy. Some office staff potentially come in to contact with a huge number of parents and children as the enter & leave the school/have enquiries whilst others may have much more admin focussed roles. Some catering staff will come into contact with all pupils who eat school dinners whilst others will work solely in the kitchen.
- Children with SEND – cannot distance and often control bodily functions or use tissues etc Ability of parents/ family to understand guidance and instructions- we have 90% EAL community and they often don’t understand the requirements- this is something we are having to support with through interpreters; Ability of families to distance and follow guidance at home – more risk that in school where we have control.
- Transport. As a City Centre school the vast majority of pupils travel to school on public transport and school transport.
- Children with SEND who do not understand the boundaries around personal space are starting to come back and they are then likely to have 1:1 contact with an adult of less than 1m for significant period of the day to keep them safe. We have segregated the toilets so that each pod has a toilet but the sinks are shared space.

1. In this and subsequent similar questions you are being asked about the typical or average behaviour and your uncertainty in this average or typical behaviour. [↑](#footnote-ref-1)
2. This question and subsequent similar questions you are asked to think about the extreme behaviours of individuals in your school. [↑](#footnote-ref-2)
3. It seems likely to us that there will be little difference in the behavior and management regime for very young children but this is an opportunity for you to disagree. [↑](#footnote-ref-3)
4. Numbers, character and organisation of year 2 to 5 (emergency workers and vulnerable children) may differ from year 6 affecting contacts. This question might not be relevant to some schools [↑](#footnote-ref-4)
